# Single-cell transcriptome sequencing reveals immunological mechanisms by which recombinant *Echinococcus granulosus* P29 protein alleviates airway inflammation in mice with allergic asthma

**DOI:** 10.1186/s13071-026-07256-w

**Published:** 2026-02-11

**Authors:** Zhichao Zhou, Jianwen Wu, Leiji Fu, Rou Wen, Xiaomin Zhang, Zexin Dang, Junyou Wu, Sijia Bao, Wenxuan Li, Mei Yin, Xiaoping Gao, Jiaqing Zhao

**Affiliations:** 1 School of Basic Medicine, Ningxia Medical University, Yinchuan, China; 2 Department of Otolaryngology Head and Neck Surgery, General Hospital of Ningxia Medical University, Yinchuan, China; 3 School of Inspection, Ningxia Medical University, Yinchuan, China; 4 Research Center for Medical Science and Technology, Ningxia Medical University, Yinchuan, China; 5 Ningxia Institute of Medical Science, Yinchuan, China; 6 The First Clinical Medical College, Ningxia Medical University, Yinchuan, China; 7 Ningxia Key Laboratory for Prevention and Control of Common Infectious Diseases, Yinchuan, China; 8 Department of Respiratory and Critical Care Medicine, Hospital of Cardiovascular and Cerebrovascular Diseases, General Hospital of Ningxia Medical University, Yinchuan, China

**Keywords:** Allergic asthma, r*Eg*.P29, Immunoregulation, Single-cell transcriptome sequencing

## Abstract

**Background:**

Allergic asthma is a major health burden. The “hygiene hypothesis” links parasitic infections to the prevention and treatment of allergic asthma. Recombinant *Echinococcus granulosus* P29 protein (r*Eg*.P29) has been shown to induce the conversion of CD4^+^ T cells to Th1 in mice, and is widely sourced for translational applications given its composition and biological safety compared with using fine-grained *Echinococcus granulosus* or antigenic extracts of cystic fluid. However, its effects on allergic asthma remain unclear. Therefore, in this study, we used experiments to investigate how r*Eg*.P29 relieves airway inflammation in mice with allergic asthma and understand the underlying immune mechanisms.

**Methods:**

Lung histiocytes from mice were examined using single-cell transcriptome sequencing in combination with experimental methods, such as flow cytometry. Serum levels of total immunoglobulin E (IgE), ovalbumin (OVA)-IgE, and inflammatory factors were measured using enzyme-linked immunosorbent assays and cytometric bead array. Finally, cellular communication analysis of target immune cells was performed using bioinformatics approaches.

**Results:**

r*Eg*.P29 significantly alleviated OVA-induced histopathological changes in the lungs of mice with allergic asthma; downregulated serum IgE levels; reduced lung tissue eosinophils, Th2, and Th17 cells; and increased Th1 and Treg cells in mouse lung tissues. The possibility of an interconversion between proliferative pathogenic Th2 cells and stem cell-like Th2 cells was noted. In Treg cells, Nr4a1 targets were detected, and a Lag3^+^Tnfrsf9^+^ Treg cell population was identified, which may play an important immunosuppressive role. Finally, CellChat analysis showed significant interactions between stem cell-like Th2 and Th1 cells, proliferative pathogenic Th2 and stem cell-like Th2 cells, and Treg and Th1 cells.

**Conclusions:**

r*Eg*.P29 can effectively alleviate OVA-induced airway inflammation in mice with allergic asthma, and multiple T cell subpopulations in lung tissues are collectively involved in the immunological effects of r*Eg*.P29.

**Graphical abstract:**

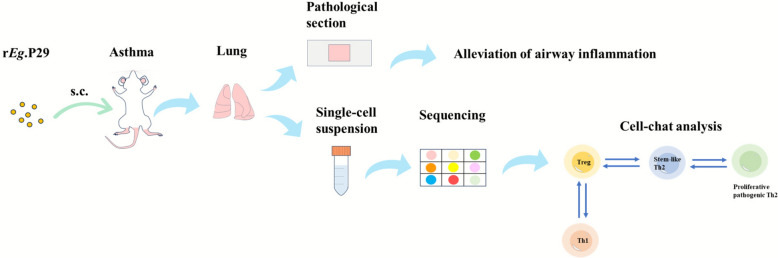

**Supplementary Information:**

The online version contains supplementary material available at 10.1186/s13071-026-07256-w.

## Background

Asthma is a common chronic disease characterized by chronic airway inflammation and hyperresponsiveness. Approximately 358 million people worldwide, particularly in developing countries [1], suffer from asthma. Patients with asthma may experience respiratory, endocrine, and cardiovascular symptoms, which impose a huge burden on both patients and society [2, 3]. The occurrence of allergic asthma, the most common type of asthma, is closely associated with the exposure to allergens, including house dust mites, pathogens, and pollen [4]. Studies have shown that in allergic asthma, there is an imbalance in Th1/Th2 cells, a large accumulation of IL-17^+^ cells and eosinophils, a reduction and impaired function of Treg cells, and an imbalance in serum immunoglobulin E (IgE) and inflammatory factors [5–8]. The current treatment for patients with allergic asthma is based on drug therapy with inhaled glucocorticoids and long-acting β2 agonists; however, increasing drug resistance remains an urgent problem that needs to be emphasized and addressed. Therefore, the development of novel therapeutic methods for patients with allergic asthma is an urgent and important research topic at present [9, 10].

Echinococcosis is a zoonotic disease caused by *Echinococcus granulosus*. According to the “hygiene hypothesis,” studies have shown that the immunomodulatory molecules in the cyst fluid of echinococcosis can suppress allergic airway inflammation by upregulating Treg cells [11]. In another study, myeloid-derived suppressor cells were significantly increased in a mouse model with fine-grained *E*. *granulosus* tapeworm infection, and these cells mainly suppressed Th2 cells [12]. The P29 protein is expressed in *E*. *granulosus*. Recombinant P29 protein (r*Eg*.P29) can promote the differentiation of CD4^+^ T cells into Th1 cells by upregulating the expression of miR-126a-5p [13]. In allergic asthma, Th2 cells accumulate in the airways and secrete large amounts of type 2 cytokines such as IL-4, IL-5, and IL-13 [14]. Moreover, r*Eg*.P29 can promote Th1 immune responses. Therefore, we speculated that r*Eg*.P29 may play a role in alleviating airway inflammation in allergic asthma by promoting a shift from Th2- to Th1-mediated immune responses.

In allergic asthma-related studies, single-cell transcriptome sequencing (scRNA-seq) has been used to understand the development of and interactions among heterogeneous immune cells and to detect the expression of genes and transcriptional isoforms at the genome-wide level [15]. Alladina et al. used scRNA-seq to demonstrate transcriptional programs and cellular circuits, such as tumor necrosis factor (TNF) family signaling, specific to allergic asthma [16]. Another scRNA-seq study using showed that IL18R1-related molecules might be important for predicting severe asthma and pulmonary fibrosis [17].

Therefore, using scRNA-seq and flow cytometry, we aimed to investigate how r*Eg*.P29 relieves airway inflammation in mice with allergic asthma and understand the underlying immune mechanisms.

## Methods

### Mouse asthma model and ethics

The BALB/c mice used in this study were purchased from Beijing Huafukang Bio-technology Co. All the experiments were approved by the Ethical Review Committee of Ningxia Medical University (approval no. 2024-N088). After 1 week of acclimatization, the mice were randomly divided into four groups: a blank control group (Con), a blank + r*Eg*.P29 intervention control group (Con + r*Eg*.P29), an OVA-induced allergic asthma model group (OVA), and an OVA-induced allergic asthma + r*Eg*.P29 intervention treatment group (OVA + r*Eg*.P29). Treatment with r*Eg*.P29 was initiated according to the following protocol: on days −1, 6, and 13, the Con + r*Eg*.P29 and OVA + r*Eg*.P29 groups were administered 100 μL of r*Eg*.P29 solution via subcutaneous multipoint injection, corresponding to 20 μg of r*Eg*.P29 protein per mouse. The Con and OVA groups were administered the same volume of phosphate-buffered saline (PBS) via subcutaneous multipoint injection in the same manner. The sensitization stage of the OVA allergic asthma model was conducted as follows: on days 0, 7, and 14, the OVA and OVA + r*Eg*.P29 groups were injected intraperitoneally with 200 μL of the OVA-induced allergic asthma model sensitization solution containing 20 μg of OVA protein and 2 mg of Al(OH)_3_ per mouse (OVA: A5503-1G, Sigma-Aldrich, Germany; Al(OH)_3_: 77161, Thermo Fisher Scientific, USA). The Con and Con + r*Eg*.P29 groups were injected intraperitoneally with the same volume of PBS in the same manner. The stimulation phase of the OVA allergic asthma model was performed as follows: from day 21 to day 27, the OVA and OVA + r*Eg*.P29 groups were administered 20 μL of OVA-stimulation solution (5 mg/mL), corresponding to 100 μg of OVA protein per mouse, via nasal instillation. The Con and Con + r*Eg*.P29 groups were administered the same volume of PBS via nasal instillation in the same manner. At the end of the nasal drip, the construction of the OVA-induced allergic asthma mouse model and treatment with r*Eg*.P29 were completed. The r*Eg*.P29 used in this study was obtained from the Ningxia Key Laboratory of Common Infectious Diseases, China [18].

### Lung tissue single-cell suspension preparation

After preparation of the allergic asthma mouse model, lung tissue samples were collected from mice in accordance with animal ethics requirements. The right lower lobes of the right lung and left lung tissues were extracted. The lung tissues were digested in a solution containing 1 mg/mL collagenase I for 1 h at 37 ℃ with shaking at 220 rpm. After removal of red blood cells by centrifugation at 350*g* using red blood cell lysis buffer on ice for 5 min (R1010, Solarbio, China), the lung tissue cells were washed and suspended to complete the preparation of a single-cell suspension. Single-cell transcriptome sequencing was performed with one sample per group, for a total of four samples.

### Flow cytometry

After preparation of the single-cell suspension from mouse lung tissue, flow cytometry was performed using the following antibodies: APC-Cy7-anti-mouse-CD45 (cat. no. 557659, 30-F11, BD, USA), FITC-anti-mouse-CD11b (cat. no. 101206, M1/70, BioLegend, USA), PE-anti-mouse-Gr-1 (cat. no. 108408, RB6-8C5, BioLegend, USA), APC-anti-mouse-CD170 (cat. no. 155508, S17007L, BioLegend, USA), APC-Cy7-anti-mouse-CD3 (cat. no. 100222, 17A2, BioLegend, USA), FITC-anti-mouse-CD4 (cat. no. 100406, Gk1.5, BioLegend, USA), APC-anti-mouse-IFN-γ (cat. no. 163513, W18272D, BioLegend, USA), PE-anti-mouse-IL-4 (cat. no. 504104, 11B11, BioLegend, USA), Bv421-anti-mouse-IL-17A (cat. no. 506926, TC11-18H10.1, BioLegend, USA), Bv421-anti-mouse-CD25 (cat. no. 102043, PC61, BioLegend, USA), PE-anti-mouse-Foxp3 (cat. no. 126404, MF-14, BioLegend, USA), APC-anti-mouse-CD223 (cat. no. 125210, C9B7W, BioLegend, USA), PE-anti-mouse-CD137 (cat. no. 106106, 17B5, BioLegend, USA), and Alexa Fluor 647-anti-mouse-Nr4a1 (cat. no. 566735, 12.14,BD, USA). Intracellular cytokine production was stimulated using Cell Activation Cocktail (with brefeldin A) (cat. no. 423304, BioLegend, USA), and intranuclear transcription factor staining was performed using True-Nuclear™ Transcription Factor Buffer Set (cat. no. 424401, BioLegend, USA) according to the manufacturer’s instructions. After staining, samples were analyzed using a BD FACSCelesta flow cytometer (BD, USA), and data were analyzed using FlowJo software (version 10.10.0).

### Histopathological examination of mouse lungs

After sampling, the upper lobe of the right lung was fixed using 4% paraformaldehyde, and mouse lung tissues were stained with hematoxylin and eosin (H&E), periodic acid–Schiff (PAS), Masson, and myeloperoxidase (MPO) according to the manufacturer’s instructions.

### Detection of inflammatory factors in mouse serum

Mouse serum levels of IL-6, IL-10, TNF, IFN-γ, and IL-17A were measured using the Mouse Th1/Th2/Th17 CBA Kit (cat. no. 560485, BD, USA) according to the manufacturer’s instructions. The results were analyzed using FCAP software (version 3.0.1).

### Chromium 10X Genomics ScRNA-seq

First, a single-cell suspension was loaded onto the 10X Chromium according to the manufacturer’s instructions for the 10X Genomics Chromium Single-Cell 3′ kit (V3), and a total of 38,802 cells were captured across the four samples. Next, complementary DNA (cDNA) amplification and library preparation were performed following standard protocols. The sequencing depth was at least 20,000 reads per cell, and the libraries were sequenced using the Illumina NovaSeq 6000 Sequencing System.

### Data analysis

The scRNA-seq data were converted to FASTQ format using Bcl2fastq software (version 5.0.1) and aligned to the reference genome using CellRanger. The resulting data were filtered, normalized, and clustered using Seurat. Cells were projected into a two-dimensional (2D) space using uniform manifold approximation and projection (UMAP). The screening criteria for marker genes were as follows: more than 10% of the cells in either the target subpopulation or control subpopulation expressed the gene; *P* ≤ 0.01; and a log-fold change of gene expression (logFC ≥ 0.26). Differential gene enrichment analysis was performed using the Gene Ontology (GO) and Kyoto Encyclopedia of Genes and Genomes (KEGG) databases.

### Statistical analysis

GraphPad Prism software (version 8.0; GraphPad software, San Diego, CA, USA) was used for statistical analysis. One-way analysis of variance was used to compare multiple data groups (ANOVA). Statistical significance was set at *P* < 0.05.

## Results

### r*Eg*.P29 can alleviate allergic airway inflammation in mice

**Fig. 1:**
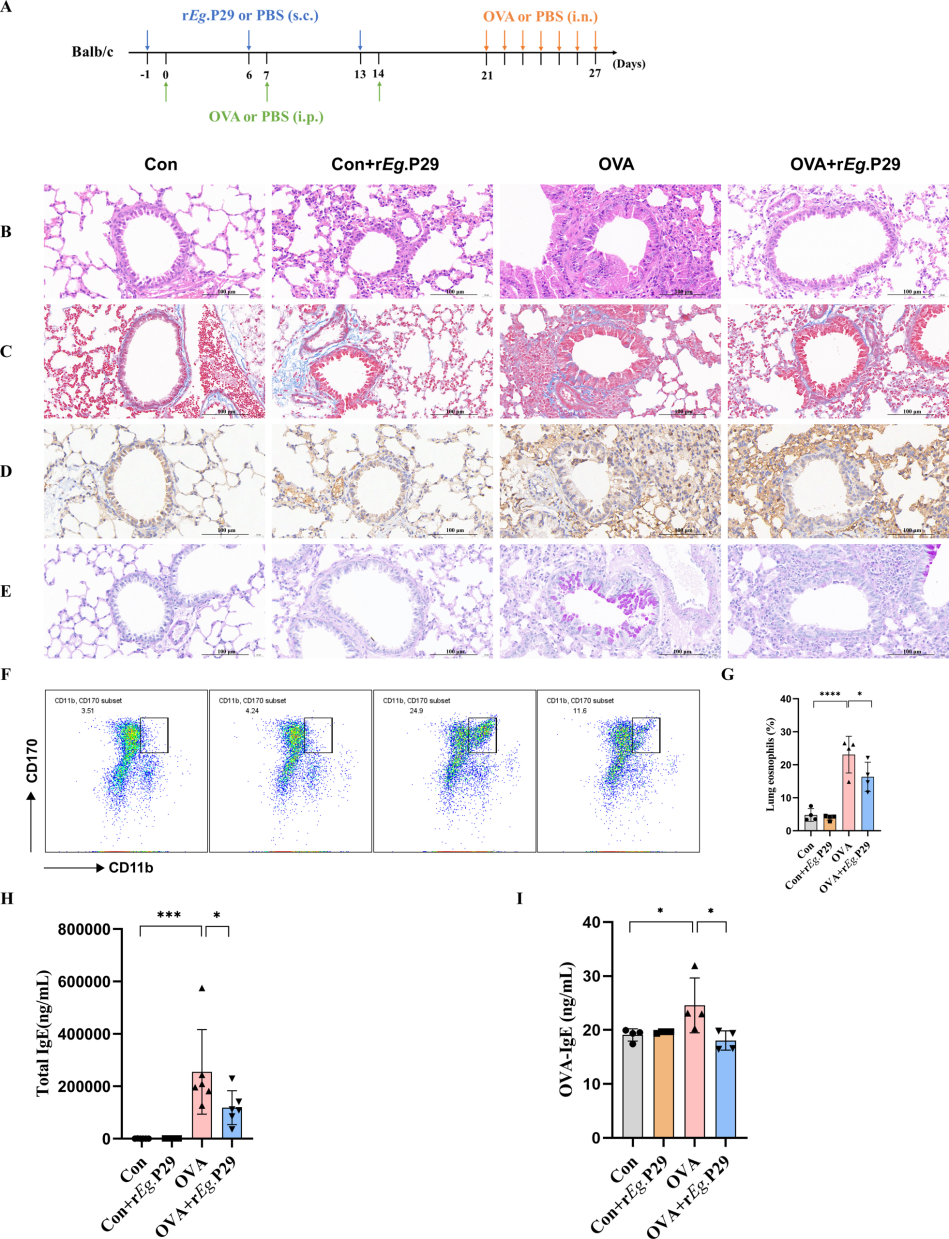
Histopathological chemical detection of mouse lung tissue, proportion of eosinophils, and detection of total serum IgE and OVA-IgE. **A** Schematic diagram of mouse allergic asthma model preparation; **B** H&E staining of lung tissue from four groups of mice; **C** Masson staining of lung tissue from four groups of mice; **D** MPO staining of lung tissue from four groups of mice; **E** PAS staining of lung tissue from four groups of mice; **F** flow cytometry scatter plot of eosinophils in lung tissues from four groups of mice; **G** statistical graph of eosinophil proportion in lung tissues from four groups of mice; **H** total serum IgE levels of four groups of mice; **I** OVA-IgE levels in serum of four groups of mice; *n* = 4; ns, no significance/no statistical difference; ^*^P < 0.05, ^**^P < 0.01, ^***^P < 0.001, ^****^P < 0.0001

Following the methods and ethical standards, an intervention treatment for airway inflammation was conducted in mice with OVA-induced allergic asthma (Fig. 1A). To visually assess whether r*Eg*.P29 could effectively alleviate airway inflammation in mice with allergic asthma, we used four histopathological chemical staining methods (H&E, Masson, MPO, and PAS) to examine mouse lung tissues. The results showed that compared with the Con group, the OVA group exhibited thickening of the airway mucosa and extensive inflammatory cell infiltration, as observed by H&E staining. Masson staining revealed extensive fibrous deposition. MPO staining showed infiltration of large numbers of inflammatory cells, and PAS staining demonstrated significant goblet cell proliferation and mucus secretion. Subsequently, compared with the OVA group, the OVA + r*Eg*.P29 group showed significant alleviation of the pathological changes mentioned above. H&E staining revealed reduced inflammatory cell infiltration in mouse lung tissue and restoration of airway thickness. Masson’s trichrome staining indicated decreased airway fibrotic deposition and attenuated pulmonary fibrosis. MPO staining was diminished, accompanied by reduced inflammatory cell infiltration. PAS staining demonstrated decreased goblet cell numbers and alleviated airway mucus secretion (Fig. 1B–E). In addition to histopathological tests, flow cytometry was used to analyze eosinophils in lung tissues. The results showed that, compared with the Con group, eosinophil levels were significantly increased in the OVA group, whereas compared with the OVA group, eosinophil levels were significantly decreased in the OVA + r*Eg*.P29 group (Fig. 1F, G). Finally, the serum levels of total IgE and OVA-IgE were measured to assess the degree of allergic inflammation. The results showed that compared with the Con group, the OVA group had significantly increased levels of total IgE and OVA-IgE, whereas compared with the OVA group, the OVA + r*Eg*.P29 group showed significantly decreased levels of total IgE and OVA-IgE (Fig. 1H, I). The above findings indicate that r*Eg*.P29 can effectively alleviate airway inflammation in mice with OVA-induced allergic asthma.

### r*Eg*.P29 effectively regulates serum cytokine imbalance in mice with allergic asthma

**Fig. 2:**
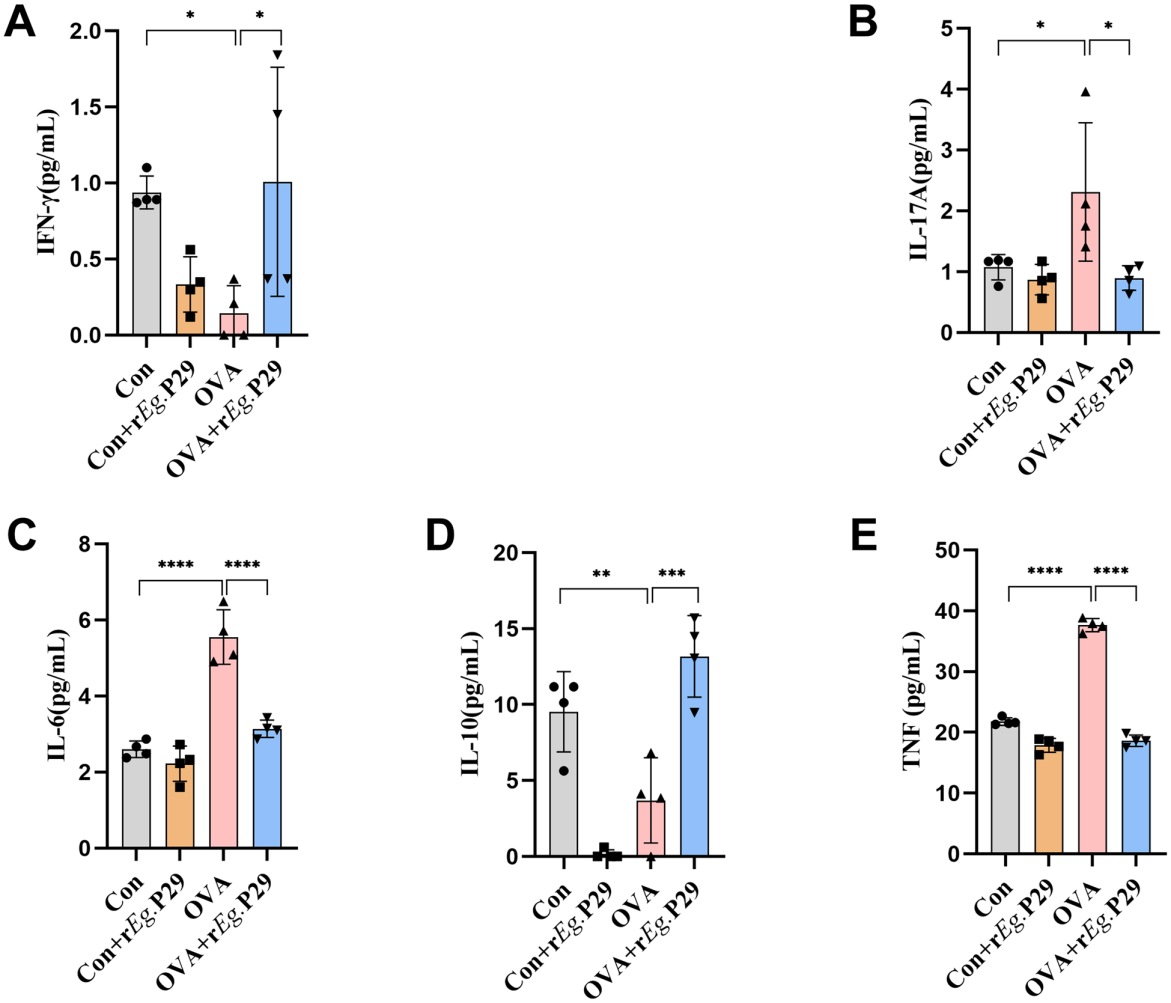
Detection of inflammatory factors in mouse serum (CBA). **A** Detection of IFN-γ level in mouse serum; **B** detection of IL-17A in mouse serum; **C** detection of IL-6 level in mouse serum; **D** detection of IL-10 level in mouse serum; **E** detection of TNF level in mouse serum. *n* = 4; ns, no significance/no statistical difference; ^*^P < 0.05, ^**^P < 0.01, ^***^P < 0.001, ^****^P < 0.0001

In addition to the immune cells being involved in the pathogenesis of allergic asthma, as mentioned above, cytokines, as mediators of immune cell functions, also play a crucial role in the r*Eg*.P29-mediated alleviation of allergic asthma in mice. We examined cytokine levels in mouse serum using cytometric bead array (CBA). The results showed that IL-6, IL-17A, and TNF were significantly upregulated, whereas INF-γ and IL-10 were significantly downregulated in the OVA group compared with those in the Con group. Meanwhile, INF-γ and IL-10 were significantly upregulated, whereas IL-6, IL-17A, and TNF were significantly downregulated in the OVA + r*Eg*.P29 group compared with the OVA group (Fig. 2A–E). These results suggest that r*Eg*.P29 can alleviate airway inflammation in mice with allergic asthma by regulating cytokine imbalance.

### Comprehensive analysis of immune cells in mouse lung tissue

**Fig. 3:**
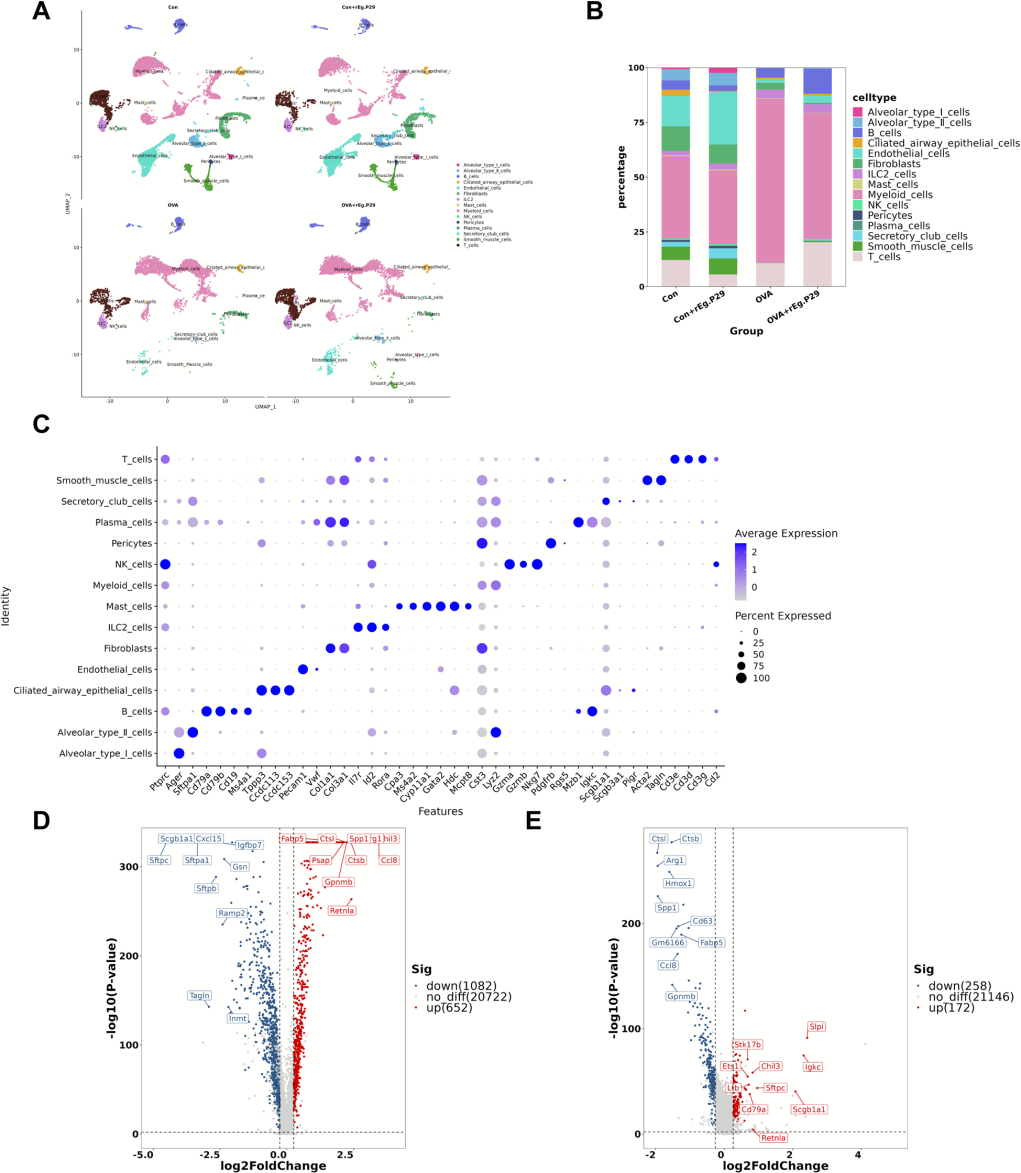
Immune panorama of the single-cell transcriptome in mouse lung tissue. **A** UMAP plot of mouse lung tissue cells; **B** proportion plot of various cell types in the lung tissues of the four groups of mice; **C** gene expression maps of various cell types in mouse lung tissue; **D** volcano plot of differentially expressed genes in mouse lung tissue cells between the Con and OVA groups; **E** volcano plot of differentially expressed genes in mouse lung tissue cells between the OVA and OVA + r*Eg*.P29 groups

To further investigate the immunological mechanism, we conducted scRNA-seq of mouse lung cells. A total of 38,802 cell profiles were obtained through scRNA-seq, including 15,562 cells in the Con group; 10,359 cells in the Con + r*Eg*.P29 group; 5807 cells in the OVA group; and 7074 cells in the OVA + r*Eg*.P29 group. After filtering out low-activity cells, 32,002 high-activity cell profiles were retained, comprising 12,645 cells in the Con group; 8785 cells in the Con + r*Eg*.P29 group; 4725 cells in the OVA group; and 5847 cells in the OVA + r*Eg*.P29 group. Highly active cells accounted for more than 80% of the total cells in each group of samples and were used for subsequent analysis (Additional File 1). After quality control, principal component analysis was performed for dimensionality reduction, and immune cells from the four groups were visualized using UMAP plots (Fig. 3A, C). Comparative analysis showed significant differences in the proportions of immune cells among the four groups (Fig. 3B). Finally, differential gene analyses across the four groups of samples revealed significant genetic difference in lung cells from mice with allergic asthma compared with those from mice in a normal physiological state (Fig. 3D, E). These findings highlight the importance of scRNA-seq analysis for exploring the immunological mechanism by which r*Eg*.P29 effectively alleviates airway inflammation in mice with allergic asthma.

### Panoramic changes in lung T cells during r*Eg*.P29 alleviation of airway inflammation in mice with allergic asthma

**Fig. 4:**
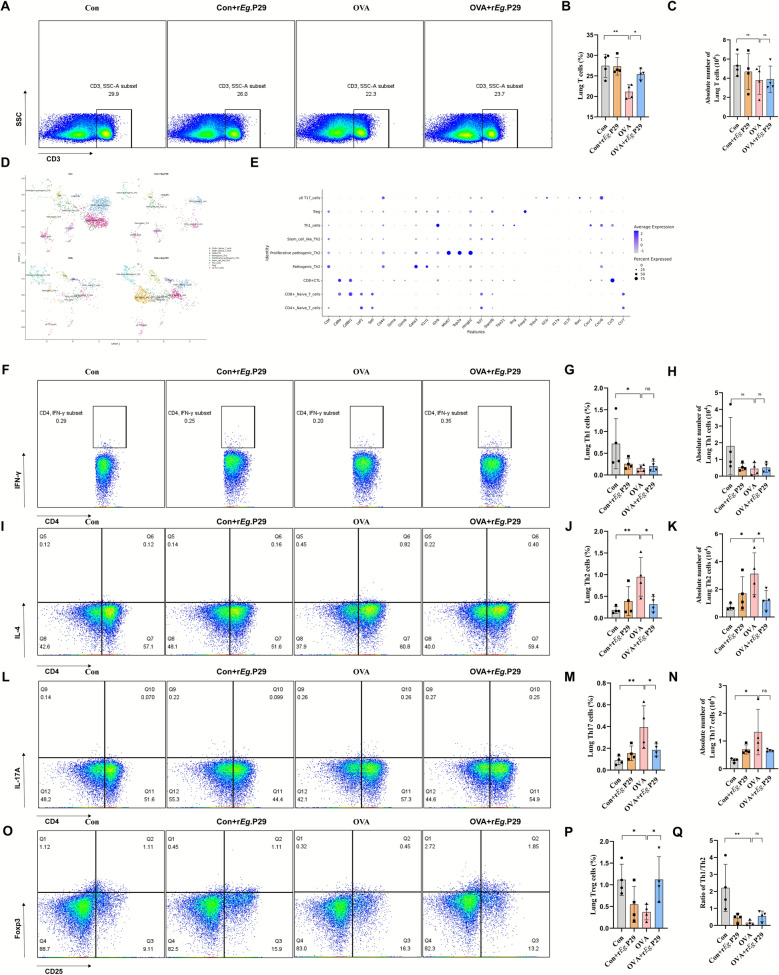
Panoramic view of T cell immunity in mouse lung tissue. **A** Flow cytometry scatter plot of total T cells in mouse lung tissue; **B** statistical chart of the proportion of total T cells in mouse lung tissue; **C** statistical chart of the absolute count of total T cells in mouse lung tissue; **D** UMAP plot of T cell reaggregation clusters from single-cell transcriptome sequencing of mouse lung tissue; **E** gene expression map of T cell reaggregation clusters from single-cell transcriptome sequencing of mouse lung tissue with cell annotations; **F** flow cytometry scatter plot of Th1 cells in mouse lung tissue; **G** statistical chart of the proportion of Th1 cells in mouse lung tissue; **H** statistical chart of the absolute count of Th1 cells in mouse lung tissue; **I** flow cytometry scatter plot of Th2 cells in mouse lung tissue; **J** statistical chart of the proportion of Th2 cells in mouse lung tissue; **K** statistical chart of the absolute count of Th2 cells in mouse lung tissue; **L** flow cytometry scatter plot of Th17 cells in mouse lung tissue; **M** statistical chart of the ratio of Th17 cells in mouse lung tissue; **N** statistical chart of the absolute count of Th17 cells in mouse lung tissue; **O** flow cytometry scatter plot of Treg cells in mouse lung tissue; **P** statistical chart of the proportion of Treg cells in mouse lung tissue; **Q** statistical chart of Th1/Th2 ratio in mouse lung tissue; *n* = 4; ns, no significance/no statistical difference; ^*^P < 0.05, ^**^P < 0.01, ^***^P < 0.001, ^****^P < 0.0001

After conducting the principal component-based dimensionality reduction analysis and annotating the cell populations, cells with high expression of *Cd3e*, *Cd3d*, *Cd3g*, and *Cd2* were identified as T cells (Fig. 3C). Comparison of T cell proportions among groups showed that the total number of T cells in the OVA group was reduced compared with that in the Con group (Fig. 3B). After treatment with r*Eg*.P29, the total T cell population in the OVA + r*Eg*.P29 group was increased, and T cell ratio trends observed by lung tissue flow cytometry (Fig. 4A–C) were comparable. T cell subpopulations play a crucial role in the pathogenesis of allergic asthma. Therefore, we performed a reclustering analysis of the T cell populations and annotated the cell populations on the basis of relevant cellular markers. The results showed that Th1 and Treg cells in the OVA group were reduced compared with those in the Con group. However, following intervention with r*Eg*.P29, Th1 and Treg cells in the OVA + r*Eg*.P29 group were increased (Fig. 4D, E). Previous studies have reported dysregulation of the Th1/Th2 ratio in allergic asthma [6, 19]. Therefore, we used flow cytometry to examine Th1, Th2, Th17, and Treg cells in mouse lung tissues. The results showed that the proportions of Th1 and Treg cells were significantly decreased, whereas those of Th2 and Th17 cells were significantly increased in the OVA group. However, in the Con group, the Th1 cell proportions were significantly upregulated, and Th2 cell proportions were significantly downregulated, that is, the ratio of Th1/Th2 cells was significantly downregulated. Compared with the OVA group, the OVA + r*Eg*.P29 group showed significantly increased proportions of Th1 and Treg cells and significantly decreased proportions of Th2 and Th17 cells, indicating an increased Th1/Th2 ratio (Fig. 4Q). The trends observed for Th1 and Treg cells by flow cytometry were consistent with the scRNA-seq described above (Fig. 4F–P). The results suggest that r*Eg*.P29 treatment reverses the Th1/Th2 cell imbalance in the lung tissues of mice with allergic asthma.

### r*Eg*.P29 may alleviate airway inflammation in mice with allergic asthma by inhibiting the remodeling of stem cell-like Th2 cells into proliferative pathogenic Th2 cells

**Fig. 5:**
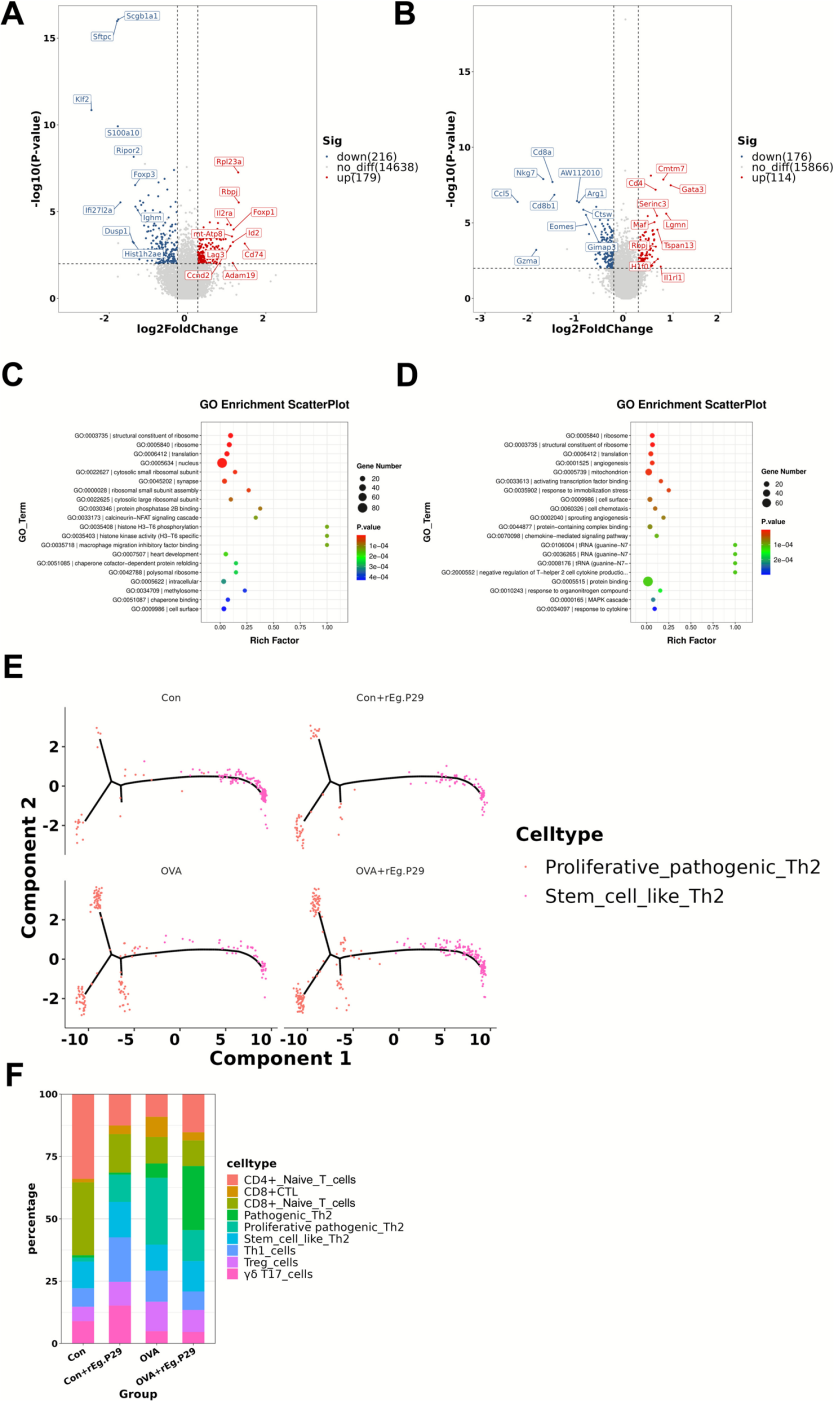
Single-cell transcriptome sequencing of mouse lung tissue for functional analysis of Th2 cells. **A** Volcano plot of differentially expressed genes between proliferative pathogenic Th2 cells OVA versus Con; **B** volcano plot of differentially expressed genes between proliferative pathogenic Th2 cells OVA + r*Eg*.P29 versus OVA; **C** GO functional enrichment analysis of differentially upregulated genes in proliferative pathogenic Th2 cells OVA versus Con; **D** GO functional enrichment analysis of differentially downregulated genes in proliferative pathogenic Th2 cells OVA + r*Eg*.P29 versus OVA; **E** mimetic time-series analysis of stem cell-like Th2 and proliferative pathogenic Th2 in lung tissue of four groups of mice; **F** proportion of T cell rearrangement class cells

Recent studies have shown that an immunological mechanism exists in patients with allergic asthma that converts stem-like Th2 cells to pathogenic Th2 cells. Cells in cluster 2 highly express *Cd4*, *Gata3*, *Tcf7* (TCF1), and *Slamf6* (Ly108) and are therefore defined as stem cell-like Th2 cells; the cells in clusters 4 and 6 highly express *Cd4*, *Gata3*, *Il4*, *Il13*, *Il2rb*, and *Il1rl1* (ST2) and are thus defined as pathogenic Th2 cells (Fig. 4D, E) [20]. Interestingly, our subsequent analysis of the highly expressed genes in the cluster 4 population showed that these cells highly expressed proliferation-related genes such as *Mki67* and *Top2a*; therefore, we redefined the cluster 4 population as proliferative pathogenic Th2 cells (Fig. 4D, E). Subsequently, we conducted a differential gene expression analysis of this group of cells (cluster 4) and found that compared with the Con group, the OVA group showed upregulated expression of *Cd74* in these cells (Fig. 5A). GO functional enrichment analysis of genes that were differentially upregulated between the OVA and Con groups revealed significant enrichment in macrophage migration inhibitory factor (MIF) binding and histone H3-T6 phosphorylation, namely, the MIF pathway and the histone H3-T6 phosphorylation pathway. The enrichment of the histone H3-T6 phosphorylation pathway indicated that these cells (proliferative pathogenic Th2 cells) underwent vigorous mitosis, further confirming that this group of cells had high proliferative capacity (Fig. 5C) [21]. Another study has shown that as a MIF receptor, CD74 in MIF-deficient mice has significantly reduced the influx of eosinophils and synthesis of leukotriene C4 caused by allergens, and the assembly of lipid bodies in eosinophils caused by MIF can be halted by blocking CD74 [22]. The OVA + r*Eg*.P29 group showed significantly downregulated *Arg1* expression compared with the OVA group (Fig. 5B), and the absence of Arg-1 expression in CD4^+^ T cells has been shown to enhance the immune response of Th1 cells [23]. Therefore, GO functional enrichment analysis of the downregulated genes between these two groups revealed enrichment of negative regulation of T-helper 2 cell cytokine production, that is, negative regulation of Th2 cytokine production (Fig. 5D). These results indicate that after treatment with r*Eg*.P29, the function of proliferative pathogenic Th2 cells had weakened. Finally, intergroup analysis of stem-like Th2 cells and proliferative pathogenic Th2 cells revealed that stem-like Th2 cells were downregulated, and proliferative pathogenic Th2 cells were upregulated in the OVA group compared with those in the Con group, whereas stem-like Th2 cells were upregulated and proliferative pathogenic Th2 cells were downregulated in the OVA + r*Eg*.P29 group compared with the those in the OVA group (Fig. 5F). Simultaneously, we found that the two cell populations differed from each other using the proposed temporal sequence analysis (Fig. 5E). Therefore, we hypothesize that r*Eg*.P29 alleviates airway inflammation in mice with allergic asthma by inhibiting the remodeling of stem cell-like Th2 cells into proliferative pathogenic Th2 cells.

### r*Eg*.P29 upregulates the expression of Nr4a1 in Treg cells in the lungs of mice with allergic asthma

**Fig. 6:**
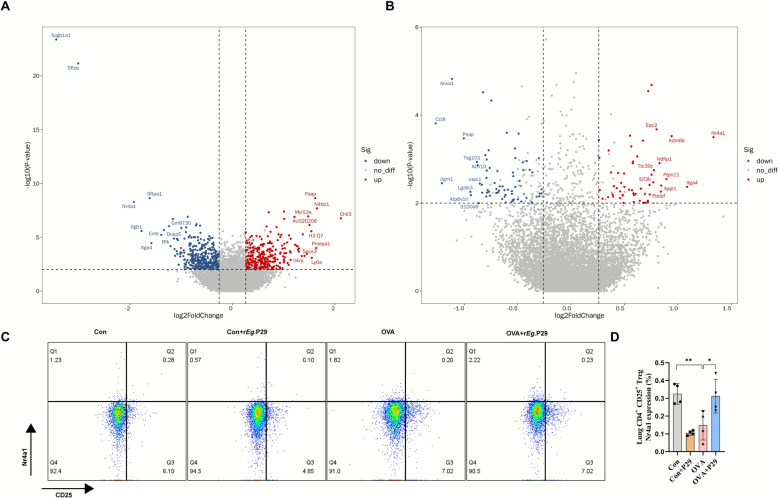
Differential gene expression analysis of Treg cells in mouse lung tissue. **A** Volcano plot of differentially expressed genes between OVA and Con in mouse lung tissue Treg cells; **B** volcano plot of differentially expressed genes between OVA + r*Eg*.P29 and OVA in mouse lung tissue Treg cells;** C** flow cytometry scatter plot of Nr4a1 expression in CD4^+^CD25^+^ Treg cells in mouse lung tissue; **D** statistical graph of Nr4a1 expression in CD4^+^CD25^+^ Treg cells in mouse lung tissue. *n* = 4; ns, no significance/no statistical difference; ^*^P < 0.05, ^**^P < 0.01, ^***^P < 0.001, ^****^P < 0.0001

Allergic asthma is a hypersensitivity disease. As immune cells that exert negative regulatory effects on immune responses, Treg cells play an important role in the regulation of allergic asthma. Our results indicate that r*Eg*.P29 can significantly increase the proportion of Treg cells. Subsequently, we conducted intergroup differential gene expression analysis of total Treg cells. Volcano plot analysis indicated that *Nr4a1* expression was significantly downregulated in the OVA group compared with that in the Con group. However, *Nr4a1* expression in the OVA + r*Eg*.P29 group was significantly upregulated compared with that in the OVA group (Fig. 6A, B). Using flow cytometry, we found that Nr4a1 expression in CD4^+^CD25^+^ Treg cells was consistent with the results mentioned above (Fig. 6C, D). Therefore, we suggest that r*Eg*.P29 may alleviate airway inflammation in mice with allergic asthma by upregulating the expression of Nr4a1 in Treg cells.

### Lag3^+^Tnfrsf9^+^ Treg may predominantly exert negative regulation in the lung tissues of mice with allergic asthma after r*Eg*.P29 treatment

**Fig. 7:**
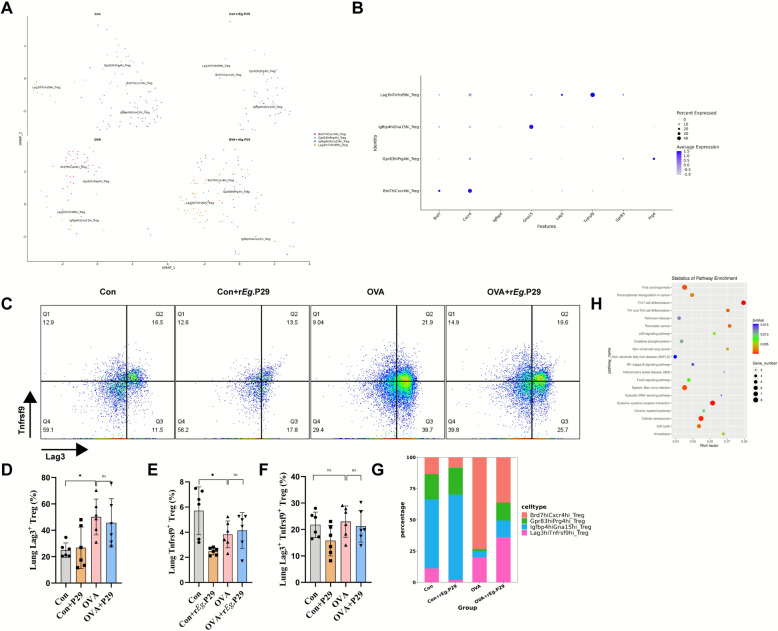
Analysis of Treg cell clustering in mouse lung tissue. **A** UMAP plot of Treg cell clustering in mouse lung tissue; **B** gene expression map of Treg cells in mouse lung tissue after clustering; **C** flow cytometry scatter plot of the expression of Lag3 and Tnfrsf9 in CD3^+^CD4^+^CD25^+^ Treg cells in mouse lung tissue; **D** flow cytometry statistical graph of the expression of Lag3^+^ cells in CD3^+^CD4^+^CD25^+^ Treg cells in mouse lung tissue; **E** flow cytometry statistical graph of the expression of Tnfrsf9^+^ cells in CD3^+^CD4^+^CD25^+^ Treg cells in mouse lung tissue; **F** flow cytometry statistical graph of the expression of Lag3^+^Tnfrsf9^+^ cells in CD3^+^CD4^+^CD25^+^ Treg cells in mouse lung tissue; **G** proportions of the four cell groups in the Treg cell clustering in mouse lung tissue; **H** KEGG functional enrichment analysis of Lag3^+^Tnfrsf9^+^ Treg cells. *n* = 6; ns, no significance/no statistical difference; ^*^P < 0.05, ^**^P < 0.01, ^***^P < 0.001, ^****^P < 0.0001

To further investigate the mechanism underlying Treg-mediated negative regulation in r*Eg*.P29 -alleviated OVA-induced allergic asthma, we conducted a reclassification analysis of the Treg cell population. Four subpopulations were identified. Cluster 0, which highly expressed *Brd7* and *Cxcr4*, was defined as Brd7^hi^Cxcr4^hi^ Treg; cluster 1, which highly expressed *Igfbp4* and *Gna15*, was defined as Igfbp4^hi^Gna15^hi^ Treg; cluster 2, which highly expressed *Lag3* and *Tnfrsf9*, was defined as Lag3^hi^Tnfrsf9^hi^ Treg; and cluster 3, which highly expressed *Gpr83* and *Prg4*, was defined as Gpr83^hi^Prg4^hi^ Treg (Fig. 7A, B). According to the existing literature, Lag3 and Tnfrsf9 are inhibitory molecules, and allergic asthma is a type of hypersensitivity reaction [24, 25]. Therefore, we analyzed cluster 2, defined as Lag3^hi^Tnfrsf9^hi^ Treg cells. To verify this group of cells, we conducted flow cytometry to detect Treg cells in the lungs of mice. The results showed that compared with the Con group, in the OVA group, the proportion of Lag3^+^ Treg and Lag3^+^Tnfrsf9^+^ Treg cells was increased, whereas that of Tnfrsf9^+^ Treg cells was decreased. Additionally, compared with the OVA group, the OVA + r*Eg*.P29 group showed no significant changes in the number of Lag3^+^ Treg cells, Lag3^+^Tnfrsf9^+^Treg cells, or Tnfrsf9^+^ Treg cells (Fig. 7C–F). The results of scRNA-seq supported the upregulation of Lag3^+^Tnfrsf9^+^Treg cells in the OVA group (Fig. 7G). A recent review indicated that Lag3 restricted the proliferation and function of Treg cells in an autoimmune diabetic mouse model [24]. Whether the upregulation of Lag3^+^ Tregs cells in the allergic asthma mouse model is related to the impaired inhibitory function of Treg cells requires further verification. Regarding Tnfrsf9, a study showed that injecting an agonistic anti-CD137 (Tnfrsf9) antibody into an allergic asthma mouse model significantly alleviated airway inflammation [25]. Finally, KEGG enrichment analysis of Lag3^+^Tnfrsf9^+^ Treg cells revealed that this subpopulation might influence the differentiation of Th1, Th2, and Th17 cells associated with the pathogenesis of allergic asthma (Fig. 7H). These results indicate that Lag3^+^Tnfrsf9^+^ Treg cells may exert contradictory effects. Therefore, it seems reasonable to explore the distinct roles of Lag3^+^ Treg and Tnfrsf9^+^ Treg cells. Thus, we hypothesize that r*Eg*.P29 alleviates airway inflammation in mice with OVA-induced allergic asthma by reducing the Lag3^+^ Treg and increasing Tnfrsf9^+^ Treg cells. However, further in-depth research and verification are required to confirm this hypothesis.

### r*Eg*.P29: alleviation of airway inflammation in mice with allergic asthma requires interactions among multiple immune cells

**Fig. 8:**
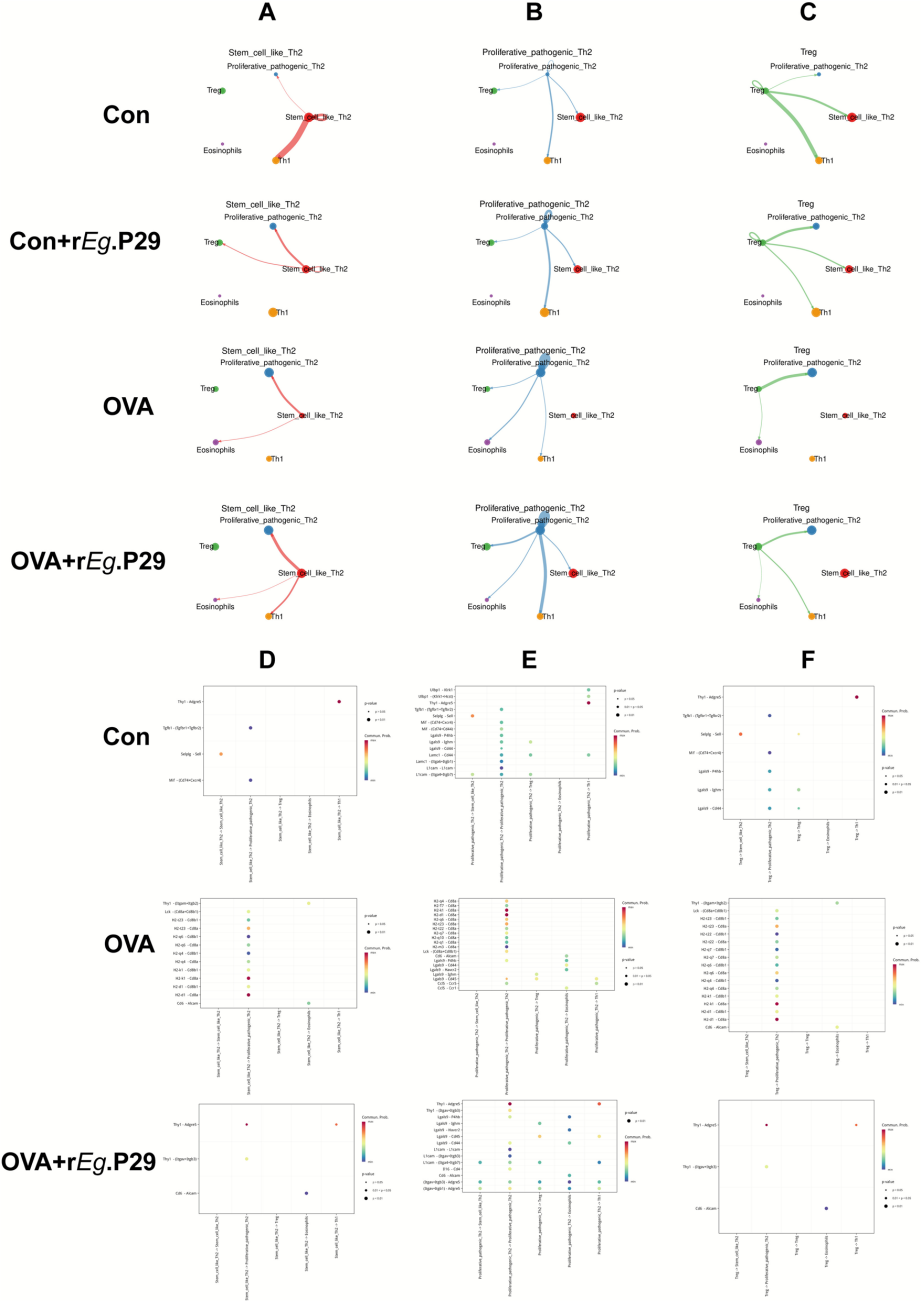
Analysis of interactions among eosinophils, Th1, Th2, and Treg cells. **A** Interactions among four groups of stem-cell-like Th2 cells; **B** interactions among four groups of pathogenic Th2 cells; **C** interactions among four groups of Treg cells; **D** interactions among three groups of stem-cell-like Th2 and Th1 cells; **E** interactions among three groups of proliferative pathogenic Th2 and stem cell-like Th2 cells; **F** interactions among three groups of Treg cells and Th1 cells

On the basis of the results provided above, we found that eosinophils, Th1, Th2, and Treg cells play roles in the r*Eg*.P29-mediated alleviation of airway inflammation in mice with allergic asthma. Subsequently, we conducted a cell communication analysis to study the mutual interactions among these cells. The results showed that, compared with the interaction between stem-like Th2 cells and Th1 cells in the Con group, these interactions were weakened in the OVA group, whereas interactions between proliferative pathogenic Th2 cells and stem-like Th2 cells were weakened. The interaction between Treg cells and Th1 cells were also weakened. Furthermore, compared with the interactions between stem-like Th2 cells and Th1 cells OVA group, these interactions had enhanced in the OVA + r*Eg*.P29 group, whereas those between proliferative pathogenic Th2 cells and stem-like Th2 cells were enhanced and those between Treg cells and Th1 cells were also enhanced (Fig. 8A–C). We conducted a detailed interaction analysis of these three types of differential cellular interactions. The results showed that the ligand–receptor pairs involved in the interactions between stem cell-like Th2 cells and Th1 cells, as well as between Treg cells and Th1 cells, were all Thy-1-Adgre5, whereas those involved in the interactions between proliferative pathogenic Th2 cells and stem cell-like Th2 cells involved the L1cam-(Itga4 + Itgb7) ligand–receptor pair (Fig. 8D–F). Compared with the Con group, the OVA group exhibited weakened interactions between Th1 cells and stem cell-like Th2 cells, proliferative pathogenic Th2 cells, and Treg cells via the Thy-1-Adgre5 ligand–receptor pair; whereas the OVA + r*Eg*.P29 group demonstrated enhanced interactions in these pathways (Fig. 8D–F). Compared with the Con group, the OVA group exhibited weakened L1cam-(Itga4 + Itgb1) ligand–receptor interactions between proliferative pathogenic Th2 cells and stem cell-like Th2, Treg cells; however, in the OVA + r*Eg*.P29 group, these interactions were enhanced (Fig. 8D–F). These results indicate that the alleviation of airway inflammation in mice with allergic asthma by r*Eg*.P29 treatment not only required the participation of multiple immune cell types but also coordinated communication among these immune cells.

## Discussion

In this study, we used r*Eg*.P29 to perform therapeutic intervention in mice with OVA-induced allergic asthma. We also analyzed the scRNA-seq of mononuclear cells from mouse lung tissue to explore the immune mechanism of r*Eg*.P29 in the treatment of allergic asthma at the molecular level. Our results indicate that r*Eg*.P29 has a therapeutic effect on airway inflammation in an OVA-induced allergic asthma mouse model. This therapeutic effect may result from the combined action of multiple immune cells and molecules, thereby providing a new theoretical basis for the prevention and treatment of allergic asthma.

The complex causes of asthma involve both genetic and environmental factors [26]. In this study, histological staining of mouse lung tissues was used to evaluate the macroscopic effects of r*Eg*.P29 on airway inflammation in mice with allergic asthma. The results showed that r*Eg*.P29 alleviated allergic asthma-induced thickening of the airway mucosa, inflammatory cell infiltration, collagen fiber deposition, goblet cell hyperplasia, and glandular mucus secretion. Eosinophils are key pathogenic factors associated with severe asthma, and detection of biomarkers such as eosinophil counts and IgE levels is crucial for defining the inflammatory characteristics in patients [27]. Therefore, in our study, we separately identified the proportion of eosinophils in mouse lung tissues and total IgE and OVA-IgE in mouse serum using flow cytometry and enzyme-linked immunosorbent assays. The results confirmed that r*Eg*.P29 could alleviate airway inflammation in mice with OVA-induced allergic asthma by downregulating eosinophils in mouse lung tissues and reducing total IgE and OVA-IgE in mouse serum. Moreover, subsequent scRNA-seq analysis further confirmed the downregulation of eosinophils in the OVA + r*Eg*.P29 group.

On the basis of these results, it can be confirmed that r*Eg*.P29 effectively alleviates OVA-induced allergic airway inflammation. Subsequently, we conducted a detailed scRNA-seq analysis. After initial dimensionality reduction and clustering of the sequencing data, 35 cell clusters were identified. After annotating the cell clusters, we found that the number of T cells was reduced in the allergic asthma model, whereas r*Eg*.P29 significantly increased the number of T cells. To verify this observation, flow cytometry analysis was conducted on lung tissues, confirming the reduction of CD3^+^ T cells in the OVA group. r*Eg*.P29 increased the proportion of CD3^+^ T cells. Subsequently, we performed a reclustering analysis of T cells and identified ten distinct cell populations. Interestingly, a recent study reported that in an allergic asthma mouse model, hypoxia-inducible factor 2α in Th2 cells can cause TCF1^+^Ly108^+^ stem cell-like Th2 cells to differentiate into ST2^+^CD25^+^ pathogenic Th2 cells, thereby exacerbating allergic asthma [20]. Through annotation of nine cell clusters within the reaggregated cells, we found that the T2 cluster cells highly expressed *Gata3*, *Tcf7* (TCF1), and *Slamf6* (Ly108) and were therefore defined as stem cell-like Th2 cells. Clusters 4 and 6 highly expressed *Il2rb* (CD25) and *Il1rl1* (ST2), and were temporarily defined as pathogenic Th2 cells. However, subsequent findings showed that cluster 4 also highly expressed proliferation genes, such as *Mki67*, indicating strong proliferative capacity. Furthermore, during immune cell communication analysis, this group of cells was found to interact more actively with other cells. Therefore, we speculated that cluster 4 cells represent the true pathogenic Th2 cells. An analysis of the intergroup cell ratio of the cluster 2 and cluster 4 cell populations showed that the OVA group exhibited a decrease in cluster 2 cells and an increase in cluster 4 cell population. In contrast, the OVA + r*Eg*.P29 group showed the opposite pattern, with increased cluster 2 cells and decreased cluster 4 cells. These results suggest that r*Eg*.P29 may alleviate airway inflammation in mice with OVA-induced allergic asthma by promoting the remodeling of pathogenic Th2 cells into stem cell-like Th2 cells. Recent studies have indicated that Th17 cells play a pivotal role in steroid-resistant asthma [28]. In allergic asthma mouse models, disease severity can be alleviated by increasing the number of Th1 cells and decreasing the number of Th17 cells in the peripheral blood of mice [29]. Our flow cytometry results also confirmed that in the OVA-induced allergic asthma group, the number and proportion of Th1 cells were decreased, whereas the proportion of Th17 cells increased. r*Eg*.P29 can inhibit airway inflammation caused by allergic asthma by reversing these changes in Th1 and Th17 cells.

On the basis of the “hygiene hypothesis,” studies have shown that the Treg response induced by parasite infection can achieve a balance with activated Th1, Th2, and Th17 cells in diseases such as allergic asthma and autoimmune disorders [30]. We inferred that r*Eg*.P29 may also exert an inhibitory effect on the type 2 inflammatory response in allergic asthma through negative immunosuppressive regulation produced by Treg cells in the body. First, we annotated cluster 7 cells and found that this group contained Treg cells. scRNA-seq and flow cytometry confirmed that Treg cells were downregulated in the allergic asthma group. After treatment with r*Eg*.P29, the quantity and proportion of Treg cells tended to increase, thereby alleviating airway inflammation in mice with allergic asthma. Second, we conducted an intergroup differential gene analysis of cluster 7 cells. Through flow cytometry and single-cell transcriptomic analysis, we confirmed that Nr4a1, located in Treg cells, was downregulated in mice with allergic asthma, whereas r*Eg*.P29 increased the expression of Nr4a1 and alleviated airway inflammation in those with allergic asthma. Studies have shown that activating Nr4a1 has therapeutic significance in lung diseases such as asthma and acute lung injury [31]. Literature reports indicate that Nr4a1 mediates *isoalloLCA*-induced proliferation of Treg cells [32]. Nr4a1 expression correlates positively with Tnfrsf9 expression on Treg cells, consistent with the findings of this study [33]. Our results confirmed this finding. Finally, we conducted a reclassification analysis of Treg cells and identified four heterogeneous Treg subpopulations. We focused on the cluster 2 subpopulation, characterized by high expression of *Lag3* and *Tnfrsf9*. Thus, Lag-3 and Tnfrsf9 may play crucial roles in Treg cell function. In summary, r*Eg*.P29 may alleviate airway inflammation in OVA-induced allergic asthma by downregulating LAG-3 and upregulating Tnfrsf9 in Treg cells. In addition to immune cell analysis, we also conducted tests on immune effector molecules, such as cytokines. Research has shown that in an allergic asthma mouse model, IL-6, IL-17A, and TNF are significantly upregulated. In contrast, the expression of the inhibitory cytokine IL-10 is significantly downregulated [34, 35]. The detection of these cytokines in mouse sera confirmed this conclusion.

Even though the present study preliminarily elucidated the important alleviating role of r*Eg*.P29 in allergic asthma, the study of proliferative pathogenic-like Th2 and Treg cells is still shallow, and its deeper immunological mechanisms need to be further investigated in the future.

## Conclusions

r*Eg*.P29 can effectively alleviate airway inflammation in mice with OVA-induced allergic asthma, and its immunological role may involve close cooperation between immune cells and immune molecules.

## Supplementary Information

Additional file 1.

## Data Availability

Data supporting the main conclusions of this study are included in the manuscript.

## Abbreviations

IgE: Immunoglobulin E
OVA: Ovalbumin
r*Eg*.P29: Recombinant *Echinococcus granulosus* P29 protein
scRNA-seq: Single-cell transcriptome sequencing
TNF: Tumor necrosis factor
PBS: Phosphate-buffered saline
GO: Gene Ontology
KEGG: Kyoto Encyclopedia of Genes and Genomes
