# Supplementary material for: Single-cell transcriptome sequencing reveals immunological mechanisms by which recombinant Echinococcus granulosus P29 protein alleviates airway inflammation in mice with allergic asthma
Source: Parasit Vectors. 2026 Feb 11;19:118. doi: 10.1186/s13071-026-07256-w (PMC12997964; doi:10.1186/s13071-026-07256-w)
Supplement: Supplementary file 1 — Additional file 1. [file 13071_2026_7256_MOESM1_ESM.docx]

| **Group** | **Number of cells before cell filtration** | **Number of cells after cell filtration** | **Percentage of cells after filtration** |
| --- | --- | --- | --- |
| Con | 15562 | 12645 | 81.26% |
| Con+P29 | 10359 | 8785 | 84.81% |
| OVA | 5807 | 4725 | 81.37% |
| OVA+P29 | 7074 | 5847 | 82.65% |
| Total | 38802 | 32002 | 82.48% |

**Data related to quality control of single-cell transcriptome sequencing in mouse lung tissue**
